# MoS_2_ QDs/8-Armed Poly(Ethylene Glycol) Fluorescence Sensor for Three Nitrotoluenes (TNT) Detection

**DOI:** 10.3390/bios11120475

**Published:** 2021-11-25

**Authors:** Xiaoyuan Zhang, Zhiqiang Su

**Affiliations:** State Key Laboratory of Chemical Resource Engineering, Beijing Key Laboratory of Advanced Functional Polymer Composites, Beijing University of Chemical Technology, Beijing 100029, China; 2020700036@buct.edu.cn

**Keywords:** hydrogel, fluorescent sensor, TNT detection, molybdenum disulfide, bionanomaterials

## Abstract

In this work, ammonia cross-linked 8-armed polyethylene glycol hydrogel material was successfully synthesized and used as a template for synthesizing nanoparticles with fluorescent properties. The 8-armed polyethylene glycol hydrogel template was used to prepare molybdenum disulfide quantum dots (MoS_2_ QDs). The ammonium tetrathiomolybdate functioned as a molybdenum source and hydrazine hydrate functioned as a reducing agent. The fluorescence properties of the as-prepared MoS_2_ QDs were investigated. The bursting of fluorescence caused by adding different concentrations of explosive TNT was studied. The study indicated that the synthesized MoS_2_ QDs can be used for trace TNT detection with a detection limit of 6 nmol/L and a detection range of 16–700 nmol/L. Furthermore, it indicated that the fluorescence-bursting mechanism is static bursting.

## 1. Introduction

Hydrogel is a polymer with a cross-linked three-dimensional network structure with hydrophilic groups. Itcan swell in water but is insoluble in water [1], and is an essential functional polymer material. Hydrogels contain hydrophilic groups that can absorb large quantities of water, which causes them to swell. Most hydrogels have a high water content, low modulus, and solid shape [2]. The multi-arm polyethylene glycol hydrogel material is a new type of polyethylene glycol hydrogel. It has various advantages that long-chain hydrogels do not have: high mechanical strength, high toughness, easy to prepare, and biocompatibility.

In recent years, molybdenum disulfide (MoS_2_) research has focused on the application of electrical and catalytic properties, and the related optical applications have not been widely studied. Since its discovery in 2004, single-atom-thick graphene has been widely used in interdisciplinary fields. Graphene materials have caused a sensation in physics, chemistry, materials science, nanoscience, engineering, and biology [3,4,5,6]. The excellent physical and chemical properties of graphene depend primarily on its inherent two-dimensional properties [7]. Many ultra-thin nanosheets of graphene-like two-dimensional inorganic materials such as MoS_2_ and tungsten disulfide are often used for cutting-edge research [8,9,10]. MoS_2_ has attracted particular attention because it can be easily peeled off. Moreover, MoS_2_ is a material with a specific energy bandgap and has high chemical and thermal stability. Its properties increase the difficulty of preparing fluorescent molybdenum disulfide materials. Therefore, it is imperative to study the synthesis method of MoS_2_ materials with a fluorescent effect.

Some fluorescent MoS_2_ materials are beginning to be fabricated. In 2010, Splendiani et al. manufactured MoS_2_ material on quartz and Si/SiO_2_ wafers using micro-lifting technology and reported the fluorescent application of this material for the first time [11]. In 2011, Coleman et al. prepared fluorescent MoS_2_ utilizing ultrasonic waves in a suitable organic solvent, which is a commercialized efficient MoS_2_ stripping method [12]. Eda et al. prepared MoS_2_ with a fluorescence effect through ultrasonic treatment and Li intercalation [13]. Stengl et al. prepared MoS2 QDs using ultrasonic treatment and liquid phase exfoliation [14]. Lin et al. prepared MoS_2_ QDs with different functionalizations through surface modification of MoS_2_ QDs with thiol-containing capping agents using a simple hydrothermal method [15]. Wu et al. [16] synthesized cysteine-functionalized MoS_2_ quantum dots (Cys-MoS_2_ QDs) by a simple amidation reaction, finding that Cys-MoS_2_ QDs had stable fluorescence. Roy et al. [17] synthesized water-soluble MoS_2_ QDs using a simplistic bottom-up hydrothermal method.

Similarly, chemical vapor deposition methods are used to synthesize MoS_2_ films with fluorescence effects on various substrates [18], and MoS_2_ films are prepared by Ar^+^ plasma irradiation methods [19]. Most of the preparation methods for MoS2 QDs are “top-down” preparation methods. These methods usually have many shortcomings, such as environmental sensitivity, massive energy consumption, the use of expensive and harmful organic solvents, and harsh pretreatment. “Bottom-up” methods such as hydrothermal synthesis have also been reported to synthesize fluorescent MoS_2_ QDs [20]. However, hydrothermal synthesis methods also have some shortcomings. For example, the synthesized products need to be separated for a long time. The reaction temperature is relatively high, and a high-pressure vessel is required. These problems may be able to be be solved by using the template method, but not many studies investigate this method.

2,4,6-Trinitrotoluene (TNT) is an important nitroaromatic explosive [21], which has explosive solid power. Because of its use in terrorist activities, a quick and straightforward technical means to detect TNT is needed. Moreover, TNT is considered a substance harmful to the environmentthat has a lasting adverse effect on the health of humans and wild animals [22]. Therefore, in the following work, a fluorescence sensor is designed to detect TNT with the fluorescence quenching effect based on MoS_2_ QDs. This work uses 8-arm polyethylene glycol hydrogel as a template material to synthesize QDs materials. Using acrylate-terminated polyethylene glycol (PEGOA) as a template, there is no need for expensive reagents, hydrothermal conditions, other mechanical equipment, and complicated post-processing procedures. The PEGOA is cross-linked by ammonia at room temperature, reduced by hydrazine hydrate, and degraded. MoS_2_ QDs with fluorescence effect will be prepared. 2,4,6-Trinitrotoluene (TNT) was selected as the target analyte. Studying the TNT concentration and the fluorescence emission intensity data of MoS_2_ QDs will provide the possible fluorescence quenching mechanism.

## 2. Materials and Methods

### 2.1. Materials

8-armed polyethylene glycol (8PEG-OH, Mw 15 KDa) was purchased from Jenkem Technologies, Plano, TX, USA. 8-armed polyethylene glycol acrylate (8PEG) was prepared using the same procedure as previously reported [23]. Polyethylene glycol diacrylate (PEGDA, Mw = 2 kDa), ammonium tetrathiomolybdate (98% purity), and TNT (1 mg/L ethanol standard solution) were purchased from Beringer (St. Helena, CA, USA). Ammonia (37% *w*/*w*) and hydrazine hydrate were purchased from the China Pharmaceutical Group (Shanghai, China). All other reagents were purchased from Aldrich and used as is unless otherwise stated. Solvents were of at least analytical quality. 

### 2.2. Synthesis of Acryloyl Chloride Modified 8-Armed Polyethylene Glycol (PEGOA)

First, 8PEG-OH (5 g) with hydroxyl capping was synthesized in a dry vacuum oven at 60 °C. 8PEG-OH was dissolved in 50 mL of Superdry solvent under nitrogen, and then the solution was placed in an ice-water bath. Then, 4 g of sodium carbonate was added to this mixture to neutralize the esterification reaction and produce the acid. The solution was stirred and reacted with a magnetic stirrer at 35 °C for 4 days under a continuous stream of nitrogen. The reacted solution was filtered through a column of alkaline alumina to remove the insoluble solids and the acid produced during the reaction. The solution was evaporated under a vacuum at 20 °C until a specific concentration was reached. An excess of cooled anhydrous ether (<4 °C) was added dropwise to the solution. The concentrated solution was collected in a centrifuge tube, stirred continuously, and centrifuged rapidly (5000 rpm, 15 min). After centrifugation was allowed to settle, the lower sediment was taken and dried in a vacuum oven at 40 °C for 6 h to obtain a white powdery solid. The dried final product was weighed, and the yield was calculated. The yield was about 72%.

### 2.3. Preparation of Ammonium Tetrathiomolybdate Solution

100 mg of ammonium tetrathiomolybdate was dissolved in 1 mL of ultrapure water to obtain an ammonium tetrathiomolybdate solution with a 0.1 mg/mL concentration. The ammonium tetrathiomolybdate decomposed easily in an aqueous solution and was easy to prepare. 

### 2.4. Synthesis of MoS_2_ QDs by 8-Armed Polyethylene Glycol Hydrogel Template Method

Three pre-prepared portions of 150 mg PEGOA were placed in 5 mL plastic centrifuge tubes. Then, 150 mL of ultrapure water was added to dissolve them. For half an hour, an ultrasonic cleaner was used for ultrasonic treatment to ensure that the solution was completely dissolved and free of inclusions or bubbles. After ultrasonic treatment, 20 μL of ammonium tetrathiomolybdate solution was added to the PEGOA solution. After evenly mixing ammonium tetrathiomolybdate and PEGOA solution, 30 μL of ammonia, 30 μL of a mixture of ammonia and hydrazine hydrate with a mass ratio of 1:1, and 30 μL of hydrazine hydrate were added to the PEGOA and ammonium tetrathiomolybdate solution. The three mixed solutions were then rapidly shaken and placed in an ultrasonic cleaner for 15 min to form the gum. 2 mL of ultrapure water was added to all three kinds of solutions and set at room temperature for four days for gel formation.

### 2.5. Instruments and Measurements

#### 2.5.1. UV-Vis Spectroscopic Testing

2 ml of the newly synthesized products of MoS_2_ QDs were added to ammonia water, ammonia and hydrazine hydrate, and hydrazine hydrate only. At the same time, 2 mL of the mixture solution of ammonium tetrathiomolybdate and 8-arm polyethylene glycol with the same concentration were added. The four solutions were diluted to about 10 mg/L with MiliQ water and put into quartz test tubes. The UV absorption spectrum was recorded at room temperature with a TU-1901 UV-Vis spectrophotometer. The test range was 200–400 nm, the scanning rate was 100 nm/min, and the aqueous solution was the reference solution.

#### 2.5.2. Dynamic Light Scattering (DLS) Testing 

The four solutions were diluted to about 2.0 × 10^−5^ mol/L with miliQ water. The particle size and distribution of the solution were analyzed by a BI-90 plus dynamic light scattering particle sizer. The particle size and its distribution were calculated with the Stokes-Einstein equation. 

#### 2.5.3. X-ray Photoelectron Spectroscopy (XPS) 

A total of 2 mL of the four solutions were refrigerated at −4 °C. They were then freeze-dried at −60 °C, 20 Pa, for 3 days. After the freeze-drying process was complete, the obtained powder was evenly applied to the conductive adhesive. The X-ray source was al Kα Ray (1486.6 eV), and the beam spot size was 400 um. The energy of full-spectrum scanning was 150 eV, and the step size was 1 eV. The ESCALAB 250 photoelectron spectrometer was utilized for the test. The test results were plotted and analyzed using Origin 8.5 software. 

#### 2.5.4. Fluorescence Emission Spectra 

At room temperature, the excitation wavelengths of four solutions were scanned with a LS-55 fluorescence spectrometer (PerkinElmer, Inc., Boston, MA, USA). The test conditions were set a slit width of 5 nm and scanning rate of 100 nm/min. We started scanning at a fixed excitation wavelength of 320 nm and calculated the Stokes displacement. Then, we gradually changed the excitation wavelength from 280 nm and obtained the corresponding emission wavelength.

#### 2.5.5. High-Resolution Transmission Electron Microscopy (HR-TEM) Measurement

The aqueous solution of PEGOA hydrogel degradation products containing MoS_2_ QDs was diluted slightly. A small amount of the solution was dropped onto the copper mesh loaded with ultra-thin carbon film with a pipette gun. The samples were allowed to air-dry overnight at room temperature. The samples were observed with a JEM-3030F high-transmission electron microscope at different magnifications. The acceleration voltage was 200 kV. There was minor damage to the material surface under this condition.

#### 2.5.6. Fluorescence Detection of TNT

The degradation products of PEGOA hydrogel MoS_2_ QDs with ammonia and hydrazine hydrate were extracted at 2 mL and diluted below the maximum detection limit of the LS-55 fluorescence spectrometer. The fluorescence spectrum test conditions in previous studies were the same. TNT solutions with different concentrations were prepared. The TNT solutions were added to the solution containing degradation products of MoS_2_ QDs with a pipette gun. The concentration of TNT solution was changed from 0 to 1000 nmol/L. The excitation wavelength of fixed fluorescence was 340 nm. The change of fluorescence intensity was measured at the emission wavelength of 416 nm. The slit width was 5 nm, and the scanning rate was 100 nm/min. 

## 3. Results and Discussion

### 3.1. MoS_2_ QDs Fabrication with the PEGOA Template Method

MoS_2_ QDs were prepared with the PEGOA template method, which is different from the MoS_2_ QDs synthesized in existing techniques. MoS_2_ QDs products were designed with PEGOA as the template and ammonium tetrathiomolybdate as the molybdenum source. The product obtained by crosslinking PEGOA with ammonia was a light yellow solution without precipitation. It took a long time to reduce ammonium tetrathiomolybdate to synthesize QDs. A group of products added to PEGOA and hydrazine hydrate were dark brown solutions and suspended brown particles. This showed that the volume-limiting effect is not strong when only in the presence of PEG molecular chains. However, its reducibility is strong, and it produces the MoS_2_ particle with an indefinite diameter. Most of the control groups without PEGOA produced black residues, indicating that ammonium tetrathiomolybdate can only be reduced to MoS_2_ precipitate insoluble in water without polymer chains. The product had no practical value. Unlike the product without the PEGOA template, the MoS_2_ QDs synthesized by the PEGOA hydrogel template were transparent solutions without residues and insoluble substances, and did not need any separation treatment. This indicates that the PEGOA hydrogel template method is a fast and straightforward method for preparing MoS_2_ QDs.

**Figure 1:**
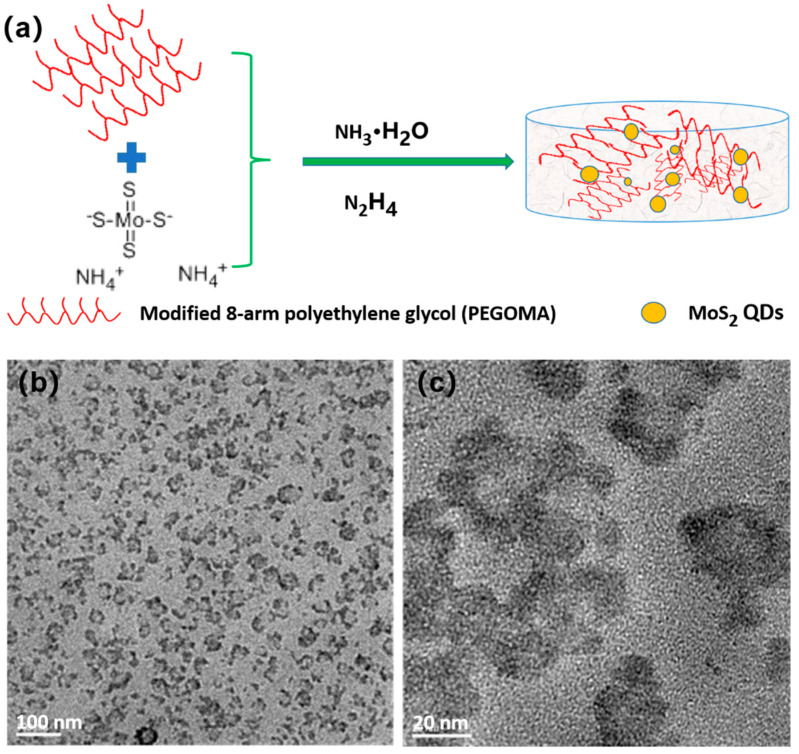
(**a**) Schematic drawing of MoS_2_ QDs formation; HR-TEM (**b**) image and (**c**) magnified image of MoS_2_ QDs synthesized by the template.

The formation of MoS_2_ QDs is schematically drawn in Figure 1a. Figure 1b,c shows that the particle size of the MoS_2_ QDs was relatively large, as observed by HR-TEM. In our previous work, the 8PEG hydrogel was used as a template to synthesize AgNCs. [24] The formation was due to the volume-limiting effect of 8PEG networks to AgNCs. The AgNCs formed inside the nanocages of the formed 8PEG gels. Results showed a homogenous distribution of AgNCs in the gel matrix. This previous work inspired us with the idea to attempt MoS_2_ QDs in situ formation. This is because the quantum dots displayed partial agglomeration, as well as becausethe core-shell structure of the synthesized QDs was caused by polyethylene glycol degradation. The existence of a single nanoparticle can still be seen from the electron micrograph with a larger magnification. Its particle size is about 10 nm, indicating that molybdenum disulfide grows within the pore size of the PEGOA gel. The formation of MoS_2_ QDs is limited in 8PEG networks, resulting in imperfect layered morphologies of MoS_2_ QDs. In addition, the formed MoS_2_ QDs are similar to nanoparticles. They are different from the layered 2D MoS_2_ QDs.

**Figure 2:**
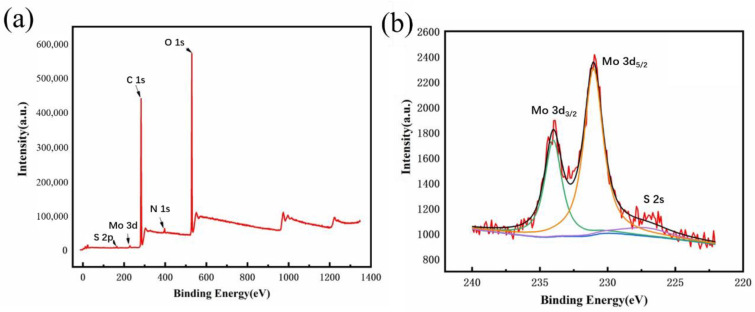
The X-ray photoelectron spectroscopy (XPS) (**a**) survey analysis and (**b**) Mo3d of MoS_2_ products.

The X-ray photoelectron spectroscopy (XPS) analysis of MoS_2_ products synthesized by the PEGOA hydrogel template method is shown in Figure 2. XPS data show that the sample contains sulfur, nitrogen, oxygen, carbon, hydrogen, and molybdenum. The presence of nitrogen indicates that ammonia successfully acts as a crosslinking agent. The sulfur element in MoS_2_ QDs prepared by the PEGOA hydrogel template method has a weak peak at 162.2 eV. The Mo3d peaks have orbital self-selected split peaks, Mo3d_5/2_ and Mo3d_3/2_, positioned around 230 eV and 234 eV in the figure, respectively. This is the characteristic peak of MoS_2_, indicating that PEGOA can be used as a template for preparing MoS_2_ QDs.

### 3.2. Photoluminescence Performance Analysis of MoS_2_ QDs

8-armed star-shaped PEG-based hydrogels (8PEG-NH3) can be cross-linked by ammonium solution 19. These were formed by an amine Michael-type addition reaction between unsaturated carbon–carbon double bonds (acrylate end groups) and reactive amine species. The degree of residual functional groups, the swelling degree, and the resulting gels’ mechanical properties can be tuned [25,26]. 

**Figure 3:**
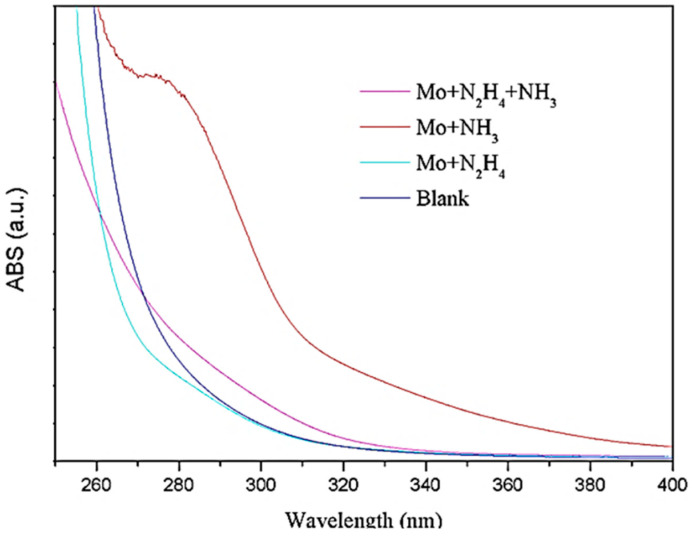
UV-Vis spectrogram of synthesized MoS_2_ QDs by the above methods.

Figure 3 shows the UV spectra of the MoS_2_ QDs synthesized by the above methods. The UV spectrum of the ammonia water group has a specific UV absorption near 285 nm. The UV absorption peak at 285 nm only appeared in the ammonia group. This is because the added ammonium tetrathiomolybdate could not be reduced entirely. The intermediate product may have a certain degree of UV absorption near 285 nm.

**Figure 4:**
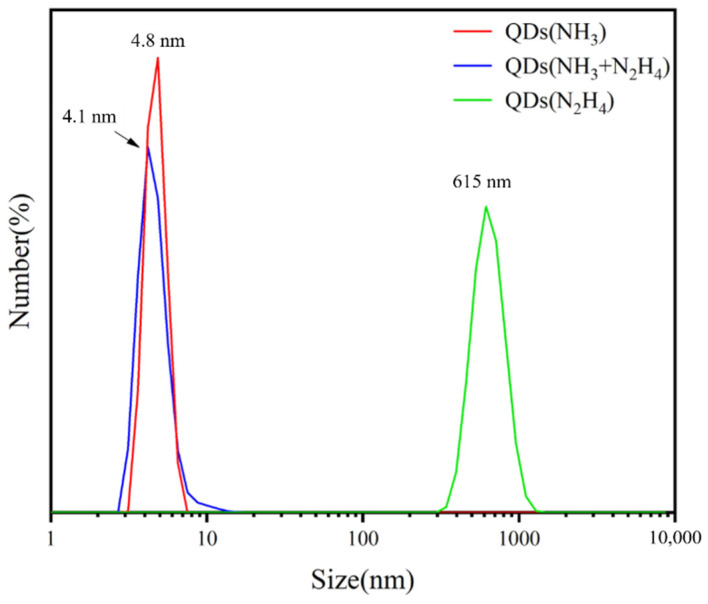
Dynamic light scattering (DLS) analysis of MoS_2_ QDs.

Figure 4 shows the dynamic light scattering (DLS) analysis of MoS_2_ QDs prepared with the PEGOA template using NH_3_, NH_3_ + N_2_H_4_, and N_2_H_4_ cross-linking agents, respectively. It can be seen from the figure that the particle size of ammonia as the additive is typically around 4.8 nm. The size of the mixture of ammonia and hydrazine hydrate is mostly about 4.1 nm. The size of hydrazine hydrate as the additive is mostly about 615 nm. This phenomenon further indicates the formation of MoS_2_ QDs in the PEGOA template.

**Figure 5:**
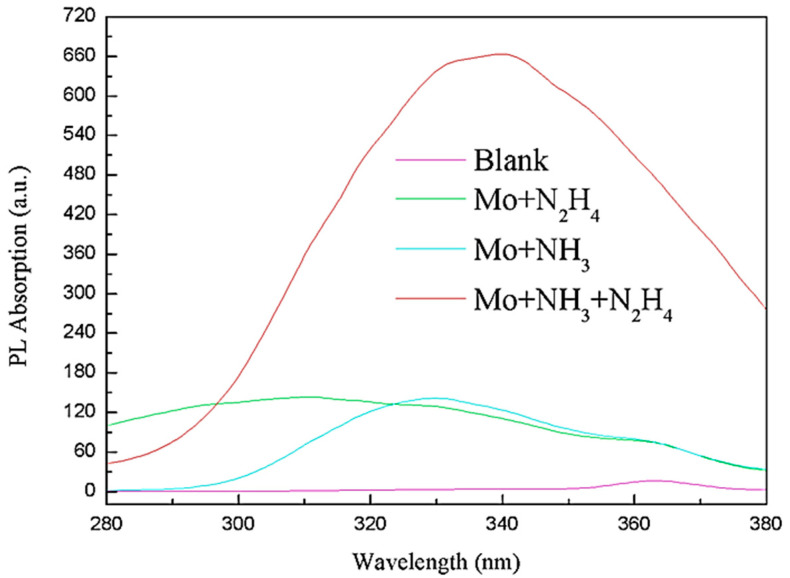
Fluorescence excitation spectrum of MoS_2_ QDs synthesized by above methods when emission spectrum is set as 420 nm.

Figure 5 shows the excitation spectra obtained by scanning the MoS_2_ QDs when the fluorescence emission spectrum was set to 420 nm. All solutions are filtered and removed through a tetrafluoroethylene filter so as not to damage the instrument while measuring the insoluble. The blank control group without PEGOA has a weak excitation peak at 365 nm, which may be the excitation peak of solvent water. The excitation peak of the MoS_2_ QDs product obtained by adding only hydrazine hydrate is shallow and wide. This indicates that the particle size distribution of MoS_2_ QDs obtained by reducing only hydrazine hydrate is wide. The width of the excitation peaks when only ammonia is added is much lower than that when only hydrazine hydrate is added. There are two little peaks at 330 nm and 360 nm, indicating that the reduction reaction occurs very slowly and only partially. The possible reason is that MoS_2_ QDs are synthesized inside the PEGOA hydrogel, and some may be synthesized outside the hydrogel, resulting in uneven particle size distribution. The ammonia and hydrazine hydrate groups show a strong excitation peak near 340 nm. The peak width was narrow, indicating that QDs with uniform particle size were synthesized inside the hydrogel.

**Figure 6:**
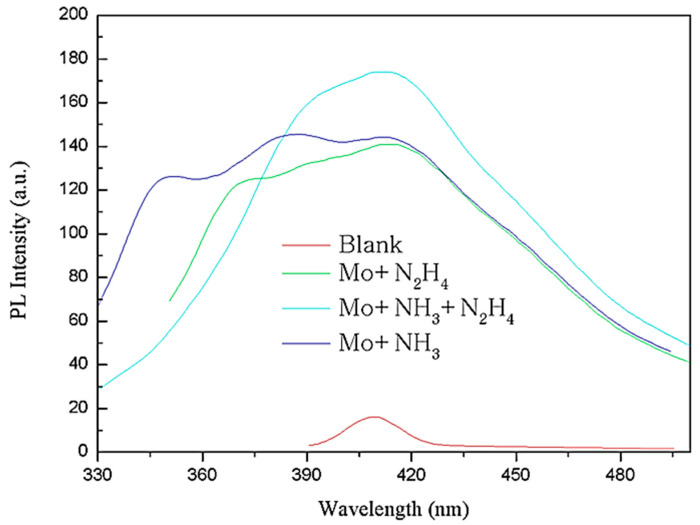
Fluorescence emission spectrum of MoS_2_ QDs synthesized by the above methods when excitation spectrum is set as 340 nm.

Since the MoS_2_ QDs have the most substantial excitation peak at 340 nm, we set the excitation light wavelength to 340 nm to detect the fluorescence emission spectra of the four groups of MoS_2_ products. The results are shown in Figure 6. The product obtained without PEGOA in the control group has a weak emission peak near 414 nm, which may be a trace amount of MoS_2_ QDs mixed with impurities. The group with only ammonia water and the group with only hydrazine hydrate showed multi-peak distribution. The peak positions ranged from 350 to 416 nm, indicating that the product’s particle size obtained without volume limitation fully reduced was unevenly distributed. The fluorescence emission peak position obtained by adding ammonia and hydrazine hydrate is at 416 nm, consistent with the results described in other documents. This is the fluorescence emission peak of MoS_2_ QDs. The peak width is narrow, indicating that particle size distribution of QD formation is relatively uniform. The Stokes shift of the obtained QDs is 76 nm, which may be one of the characteristics of QDs synthesized by the template method.

**Figure 7:**
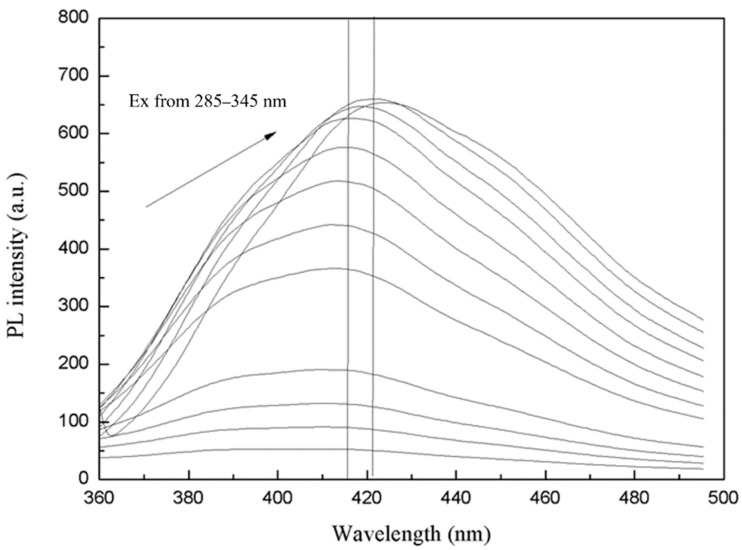
Emission spectrum of MoS_2_ QDs changes along with excitation spectrum.

Studying the position and intensity change of the emission peak of the MoS_2_ QDs can better explain the difference in the particle sizes of the synthesized QDs. As shown in Figure 7, when the excitation wavelength is increased from 285 nm to 345 nm, the peak position of the emission spectrum changes between 416–421 nm. Moreover, the peak position of the emission spectrum changes only 5 nm when the excitation wavelength changes by 60 nm. This further illustrates the advantages of the template method, which are particle size control and narrow distribution. When the peak is more significant than 345 nm, the emission spectrum drops quickly, indicating that the fluorescence excitation peak of the prepared QDs is at 340 nm. It can be seen that a slight red shift of the fluorescence emission appears with the excitation wavelength. The redshift or change in the maximum intensity indicates a change in fluorescence properties, resulting from an interaction between the 8PEG and MoS_2_ QDs. It is suggested that the change in fluorescence is due to the further growth of MoS_2_ QDs.

### 3.3. MoS_2_ QDs for Fluorescence Detection of TNT

**Figure 8:**
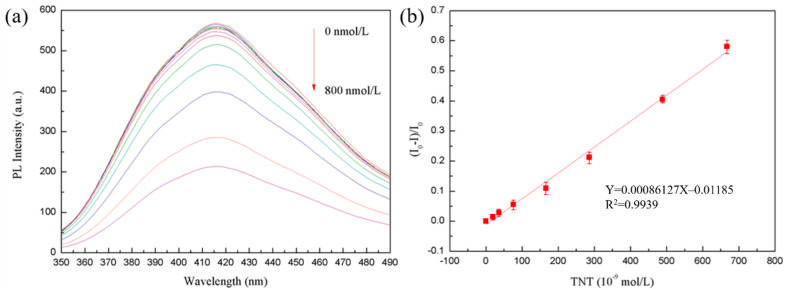
(**a**) Intensity of emission spectrum of MoS_2_ QDs change with TNT concentration. (**b**) Detection range of MoS_2_ QDs synthesized by the PEGOA template.

In Figure 8a, when a certain amount of TNT solution is added to the aqueous solution of MoS_2_ QDs, the fluorescence emitted by the MoS_2_ QDs is absorbed by the TNT molecules. This phenomenon can be attributed to fluorescence resonance energy transfer, resulting in the fluorescence quenching effect. The detection limit of the MoS_2_ QDs prepared by the template method is 8 nmol/L. This value is determined by adding a certain amount of TNT solution, and the fluorescence quenching intensity is more significant than the three standard deviations detected by the instrument [27].

Figure 8b showed the fluorescence quenching degree (the ratio of fluorescence intensity after adding TNT to solution without quencher) of MoS_2_ QDs. The fluorescence intensity is used as a characterization of the degree of fluorescence quenching. It can be seen that the fluorescence quenching degree (I_0_ − I)/I_0_ has a linear relationship with the concentration of TNT solution. In the range of TNT concentration of 16–700 nmol/L, the linear correlation coefficient is 0.9939, and the linear equation is Y = 0.00086127X − 0.01185. This indicates that MoS_2_ QDs can be used as a fluorescence detection method to detect TNT in the linear range. The detection range is 16–700 nmol/L. 

The quenching mechanism of TNT can be determined according to the following Lineweaver-Burk equation:

(1) $$1/(F_{0}-F)=1/F_{0}+K_{LB}/\left(F_{0}C_{q}\right)$$

where *F*_0_ and *F* are the fluorescence intensity of QDs when TNT is not added and when TNT is added, respectively. KLB is the corresponding static quenching constant, and *C_q_* is the concentration of the quencher. It can be seen from the formula that if (*F*_0_ − *F*)/*F*_0_ is proportional to the concentration of the liquid, the mechanism of fluorescence quenching is static quenching. Compared with the research of Xu et al., the detection limit of the sensor in this paper is reduced, which is of great significance to the improvement of the detection sensitivity of TNT [28,29]. In this work, the mechanism of sudden extinction is the coordination structure formed between MoS_2_ QDs and TNT molecules. The TNT molecules absorb the fluorescence emission of MoS_2_ QDs, which results in fluorescence quenching.

## 4. Conclusions

In summary, a novel ammonia cross-linked acrylate-terminated polyethylene glycol (PEGOA) hydrogel template was synthesized and used to prepare the nanocrystalline material MoS_2_ QDs. MoS_2_ QDs prepared by the template method have a controllable particle size, narrow distribution, and stable fluorescence. They were influenced by the different components of the incorporated cross-linking and reducing agents. The burst effect of adding different concentrations of explosive TNT to the synthesized quantum dots on the fluorescence was investigated. This investigation indicated that the synthesized MoS_2_ QDs can be used for trace TNT detection with a detection limit of 6 nmol/L and a detection range of 16–700 nmol/L. In the future, the quantum dots prepared by the template method could be used in the fields of catalytic synthesis, cellular fluorescence tracing, and fluorescence detection.

## Data Availability

Not applicable.
